# Morphological, agronomical, physiological and molecular characterization of a high sugar mutant of sugarcane in comparison to mother variety

**DOI:** 10.1371/journal.pone.0264990

**Published:** 2022-03-10

**Authors:** Qaisar Khan, Ying Qin, Dao-Jun Guo, Xiu-Peng Zeng, Jiao-Yun Chen, Yu-Yan Huang, Quang-Kiet Ta, Li-Tao Yang, Qiang Liang, Xiu-Peng Song, Yong-Xiu Xing, Yang-Rui Li

**Affiliations:** 1 College of Agriculture, Guangxi University, Nanning, China; 2 Guangxi Key Laboratory of Sugarcane Genetic Improvement, Key Laboratory of Sugarcane Biotechnology and Genetic Improvement (Guangxi), Ministry of Agriculture and Rural Affairs, Sugarcane Research Institute of Guangxi Academy of Agricultural Sciences, Sugarcane Research Center of Chinese Academy of Agricultural Sciences, Nanning, China; National Bureau of Plant Genetic Resources, INDIA

## Abstract

Sugarcane is a significant crop plant with the capability of accumulating higher amount of sucrose. In the present study, a high sucrose content sugarcane mutant clone, GXB9, has been studied in comparison to the low sucrose mother clone B9 on morphological, agronomical and physiological level in order to scrutinize the variation because of mutation in GXB9 in field under normal environmental condition. The results showed that GXB9 has less germination, tillering rate, stalk height, leaf length, leaf width, leaf area, number of internodes, internode length and internode diameter than B9. Qualitative traits of leaf and stalk displayed significant variation between GXB9 and B9. Endogenous hormones quantity was also showed variation between the two clones. The relative SPAD reading and chlorophyll a, b concentrations also showed variation between GXB9 and B9. The photosynthetic parameter analysis indicated that the GXB9 has significantly higher net photosynthetic rate (Pn), stomatal conductance (gs) and transpiration rate (Tr) than B9. The qRT-PCR analysis of genes encoding enzymes like *SPS*, *SuSy*, *CWIN*, and *CeS* showed upregulation in GXB9 and downregulation in B9. However, these genes were significantly differentially expressed between the immature and maturing internodes of GXB9. The cane quality trait analysis showed that GXB9 had higher juice rate, juice gravity purity, brix, juice sucrose content and cane sucrose content than B9. The yield and component investigation results indicated that GXB9 had lower single stalk weight, however higher number of millable stalks per hectare than B9, and GXB9 had lower theoretical cane yield than B9. SSR marker analysis showed genetic variation between GXB9 and B9. This study has shown significant variation in the traits of GXB9 in comparison to B9 which advocates that GXB9 is a high sugar mutant clone of B9 and an elite source for future breeding.

## Introduction

Sugarcane (*Saccharum* spp. hybrid) is an important sugar and bioenergy crop because of its capability for high sucrose content accumulation in stalks; chiefly in ripen internodes [1–3]. Sugarcane is a C_4_ photosynthetic plant with good competence to use the resources, and gives efficient production of products particularly sucrose [4–7]. China is one of the main sugar producers in the world while Guangxi is the largest sugarcane and sugar-producing area in China, whose sugar production accounts for more than 60% of the total in China [8]. Sugar yield is a quantitative character which depends upon cane yield, sucrose content and sugar recovery [9].

The sugarcane growth cycle passes through different phases, including germination phase, tillering phase, grand growth phase, maturity and ripening phase from planting to harvesting. Sugarcane plants are perennial, whose stalks can be several meters in length having juicy with higher concentrations of sucrose. Sugarcane root system consists of adventitious and permanent shoot root types. Stalk morphology is highly variable from one genotype to another and represents an important element for varietal characterization [10]. Sugarcane leaves, which consist of leaf blade and sheath, are numbered from top to bottom starting with the uppermost leaf showing a visible dewlap designated as leaf +1 (2). Leaf traits including the shape, size, distribution of trichomes, shape of the ligule and auricles are of taxonomic importance for varietal identification [10].

Sucrose is manufactured in the green leaves of sugarcane by the process of photosynthesis, transported through phloem and unloaded into the sink for metabolism and accumulation. Sucrose accretion in sugarcane stalks is influenced by sucrose supply, metabolism and sink strength (3). The activity of photosynthesis in the green leaves determines the quantity of sucrose to sink organs. A network of enzymes such as sucrose phosphate synthase (SPS), sucrose synthase (SuSy) and invertases manages the sucrose metabolism and storage in the internodes of sugarcane plants [11, 12]. Many other factors such as cellulose, sugar transporter, transcription factors, protein kinases, and hormones signaling also play roles in sucrose synthesis, transportation and accumulation in sugarcane stalks [13–15]. The genes allied with sucrose metabolism and source-sink signaling transduction have been well studied in plants [14, 16]. However, the mechanism of molecular regulation of sucrose storage in sugarcane is not well understood yet.

Sugarcane breeding programs have faced several challenges due to narrow genetic background, and only a few ancestral clones have been used in the hybridization schemes. Additionally, this problem has been intensified as only few well-liked sugarcane hybrids have been broadly utilized as parental lines [17–19]. For instance, over 90% of sugarcane cultivars in America could be found back to 10 hereditary clones [20]. The cultivar ROC22 has covered about 50–60% of cultivation areas in China in past 15 years, and has been the most regularly adopted parental genetic source for breeding programs [21, 22]. Hence, expanded genetic base for sugarcane cultivars would advance cane yield and decrease seriously upsetting disease eruptions because of planting varieties with diverse background in big areas. Introducing and using exclusive sugarcane clones from other countries or areas can meaningfully accelerate the attainment of sugarcane breeding goal for two important reasons, first is the top sugarcane clones, resulting from diverse breeding programs expectedly have unlike genetic backgrounds, while second is that these top sugarcane clones have high yield, high sugar and improved vital agronomic characters [23].

Genetic improvement of crops is the key element of efforts to address pressures on the worldwide food security and nutrition [24]. Recently, the somatic embryogenesis achievement through tissue culture has an excessive capacity for propagation at fast frequency [25]. It has been stated that all the redeveloped plants from the tissue culture are same as their parents. However, phenotypic discrepancies have generally been detected between regenerated plants which are associated with genetic variations in plants. These deviations might be resulted from mutations, epigenetic changes or a combination of both processes.

All the heritable variations in the nucleotide sequences or chromosomes of living organisms of a species or any other hierarchy are considered mutation. Generally, mutations maybe spontaneous due to result of errors in DNA replication which could be beneficial, harmful, or induced mutation because of exposure to radiation, chemicals, viruses, or other mutagenic agents [26]. Mutation is the final source of entire genetic vicissitudes which prepare raw material for evolution, and it is an important approach for upgrading the economic traits of crop plants [27, 28]. It has been found that the history of mutation in plants is very old, even it could be traced to 300 BC with literature of mutant crops in China [29, 30]. Few decades earlier in China, the sugarcane variety ROC22 was widely cultivated because of better quality traits, like drought resistance, higher cane yield, and sucrose content but long time cultivation declined its traits along with drought resistance [31]. The callus of cultivar ROC22 was mutagenized chemically and mutant lines were found to be suitable for upcoming drought resistance breeding [32]. Three sugarcane cultivars, NIA-0819, NIA-98 and BL4, in Pakistan were subjected to mutation through gamma radiation and the obtained mutants were with altered stomatal characteristics which positively added to yield and yield related traits in sugarcane [33]. The majority of mutations have neither negative nor positive effects on the organism in which they take place such mutations are called neutral mutations. However, some mutations have a significant positive effect on the organism in which they occur and are termed beneficial mutations. They introduce new versions of proteins that facilitate organisms to adapt changes in environment to compete the circumstances. So, beneficial mutations are essential for evolution to occur. Harmful mutations can lead to several problems particularly genetic disorders.

The objective of current research was to study the dissimilarities of a high sucrose content sugarcane mutant GXB9 on morphological, agronomical, physiological and molecular bases in comparison to low sucrose content B9 mother clone. B9 was brought from Brazil to China in October 1999, which has good characteristics like vast adaptability, good emergence rate, high tiller numbers, large number of millable canes, medium to large stalks, good defoliation and high production, however relatively lower sugar content (Wang et al. 2005). On 15th October 2013, a high sugar content clone was identified in a population of B9 and it was given name as Guixuan B9 (GXB9). After that, for several years field trials were carried out to compare the performance of GXB9 in comparison to B9 mother clone. The performance data displayed that both clones have apparently similar morphological appearance, however consistently significant difference in sugar content in cane [34]. So, it was considered that GXB9 clone has mutated, and a systematic study was planned to observe the variation in morphological, physiological, and agronomic traits of GXB9 mutant in comparison to low sucrose B9 mother clone in field under normal environmental conditions. This is the first systematic comparative study of high sucrose content GXB9 on morphological, agronomical, physiological and molecular level in comparison to low sucrose B9 mother clone. Moreover, SSR marker-based polymorphism analysis was also conducted to explore the possible genetic variation between GXB9 and B9.

## Materials and methods

### Plant material

In current study, a high sucrose sugarcane mutant GXB9, low sucrose mother clone B9 and GT32 variety were used as materials for experiment. These plant materials were provided by Sugarcane Research Institute, Guangxi Academy of Agricultural Sciences, Nanning, Guangxi, 530007, China. A total of 5 years of continuous field experiments were conducted from 2016 to 2020 to investigate the sucrose content and other cane quality traits of GXB9 mutant clone in comparison to B9 mother clone. The experiments from 2016/17 to 2019/20 were conducted in Dingdang experiment base of Sugarcane Research Institute, Guangxi Academy of Agricultural Sciences in Longan County (23° 13’ N 107° 98’ E), and the experiment in 2020/21 was conducted in the experimental field of College of Agriculture, Guangxi university in Nanning City (22° 51’ N 108° 17’ E).

At the experimental sites, soil was well prepared and 700 kg/ha compound fertilizer (N-P_2_O_5_-K_2_O: 22-8-12) were applied to the soil before planting the sugarcane setts. 10-meter-long furrows with row spacing 1.2 m were created and double bud setts were planted in double lines with a spacing of 10 cm between the lines in early March every time. The sugarcane buds planting density was 95634 buds/ha and the cane bud setts were treated with carbendazim fungicide for one hour before putting into furrow. After covering the buds with 9–10 cm of soil layer, the furrow soil surface was mulched with plastic film.

### Observation on all growth phases

After planting, the field was regularly visited to observe the plant emergence status. All the numbers of germinating shoots throughout the germination phase were counted and general health conditions of newly emerged young plants were investigated. During tillering phase, the tiller numbers and total numbers of plants were counted. During the entire growth season, the plant health condition was closely monitored. The symptoms of mosaic, smut, red rot and pokkah boeng diseases, stem borers, ants, etc. were mainly target of observation under normal field conditions.

### Observation of morphological traits

For the observation of qualitative morphological traits, twelve sugarcane plants of ten months age were randomly selected from each clone. The important morphological traits, including leaf shape, leaf color, leaf blade shape, number of green leaves, spine on the back of leaf, leaf sheath waxiness, dewlap colour, leaf sheath spines, leaf sheath color, auricle shape, hairs tufts on leaf collar, ligule shape, bud shape, bud groove, bud position, bud tip cross growth zone, bud cushion, bud tip cross growth zone, internode color, internode shape, growth rings, stalk ivory marks, root zone, waxy bands, internode alignment, bud flange, node swelling, pith, root eyes arrangement, and internode wax layer, were observed.

### Investigation of agronomical traits

At maturing phase, several important agronomical traits, such as stalk height, stalk diameter, internode length, number of internodes, single stalk weight, millable stalks/ha, bud height, bud width, leaf area, leaf length, leaf width, juice content, juice gravity purity, brix, sucrose in juice, sucrose in cane, sucrose in bagasse, were determined. Non-destructive method was followed to measure the leaf area and other characteristics of leaf using CI-203 handheld laser leaf area meter (CID Bioscience, USA). The leaf dimensions were measured during the tillering, grand growth and maturity phases of plants. The brix and sucrose content in cane were tested three times in the field as well as in the laboratory at maturing and maturation phases of each crop. Every time six healthy plants were chosen randomly from each clone and subjected to target trait measurements. Field brix was measured using handheld refractometer (ATAGO-China: http://www.atago-china.com/). Brix, juice content, bagasse moisture content, bagasse sucrose content, cane sugar content, and juice sugar content were measured using Polartronic M 202 TOUCH (589 + 882 nm: SCHMIDT+HAENSCH GmbH & Co., Berlin, Germany) equipment.

### Analyses of physiological traits

Various physiological traits, including gene expression of enzymes such as sucrose phosphate synthase (SPS), sucrose synthase (SuSy), cell wall invertase (CWIN) and cellulose synthase (CeS), photosynthetic parameters like net photosynthetic rate (Pn), stomatal conductance (gs), intercellular CO_2_ concentration (Ci), transpiration rate (Tr), SPAD (Soil plant analysis development), leaf chlorophyll content (a, b), and endogenous hormones such as indole acetic acid (IAA), gibberellic acid (GA), cytokinin (CYT) and ethylene (ETH), were determined at the different growth phases.

### SPAD

Leaf relative chlorophyll SPAD readings were taken during the tillering and grand growth phases for a total of four times. The SPAD readings were recorded on total six leaves (+1) of six sugarcane plants randomly chosen from each clone every time by using chlorophyll meter SPAD-502 (Konica Minolta Co., LTD., Osaka, Japan). SPAD readings were taken on the adaxial leaf surface which was completely exposed to sun light in a sunny day between 10:00 to 14:00 h with a temperature range 25–30°C, and three readings were recorded at each leaf avoiding from the midrib.

### Leaf chlorophyll content

The leaf samples for chlorophyll analysis were collected during the grand growth stage. Leaf (+1) was used as sample from six randomly selected plants of each clone and samples were prepared in triplicates. Chlorophyll a, b were assessed by dimethyl sulphoxide (DMSO) [35]. The UV-1800 Shimadzu spectrophotometer (Cole-Parmer Ltd., UK) was used for absorbance of wavelength (663, 645 nm) measurement.

### Photosynthetic parameters

During the tillering and grand growth phases from mid-May to August, Pn, gs, Ci and Tr in leaf were analyzed on sunny and well shining days from 10:00 to 2:00 h. The measurements were repeated two times by using LCPro-SD (ADC BioScientific UK) portable photosynthesis system. During the photosynthesis measurement, the photosynthetically active radiation in the leaf chamber, provided by leaf chamber fluorometer (LCF) light source, was adjusted to 1600 μmol m^–2^ s^–1^, relative humidity was adjusted to near ambient level 70–80%, leaf chamber CO_2_ concentration was set to 380 ml L^–1^, the air flow rate to sample cell was fitted to 400 μmol s^–1^, and chamber temperature was similar to ambient air temperature according to [36].

### Endogenous hormone quantification

For the comparative quantification of endogenous hormones such as IAA, GA, CYT and ETH, samples were collected at the tillering, grand growth and maturing phases respectively. Samples of top visible dewlap (TVD) leaf (leaf +1) were taken in triplicates for endogenous hormones quantification. The leaf samples were immediately shifted to liquid nitrogen and stored at -80°C for further analysis. For extraction of hormones, two grams of sugarcane leaf samples were weighed, and ground under liquid nitrogen using a pestle and mortar. The powdered samples were shifted to centrifuge tube, and 700 μL PBS buffer was added. The mixture was centrifuged at 12,000 rpm for 12 min at 4°C, and the supernatant was collected in Eppendorf tube. Measurements of entire hormones concentrations were carried out by using plant enzyme-linked immunosorbent assay (ELISA) kits (Wuhan Genemei Biotechnology Co., Ltd., China) according to the guidance of manufacturer and OD absorbance at 450 nm was measured with Multiskan Spectrum Microplate Spectrophotometer (Thermo Scientific, USA).

### Genes expression analyses by using qRT-PCR

During sugar accumulating and maturing phases, the immature internodes 5, 6 and maturing internodes 13, 14, were chosen as sample from six randomly selected plants of each sugarcane clone in the middle of October, November, and December. For the preparation of samples, the selected internodes were isolated from main stalk and cleaned with wet tissue paper. Hard rinds of internodes were removed and internodes chopped into small pieces, enclosed in platinum foil, immediately dipped in liquid nitrogen, and finally stored in a -80°C freezer for further analysis.

The relative expression of selected genes coding for SPS, SuSy, CWIN and CeS enzymes in the immature and maturing internodes were analyzed according to [37].

Total RNA was extracted from the samples by using TRIzol^®^ Reagent (Plant RNA purification reagent for plant tissue) according the manufacturer’s directions (Invitrogen, Carlsbard, CA, USA). First strand cDNA was created from 1 μg of extracted RNA using First Strand cDNA Synthesis Kit (Vazyme Biotech Co., Ltd., Nanjing, China) following the guidance of manufacturer. The qRT-PCR reaction was performed in a volume of 20 μL including 2 μL of cDNA, 10 μL of 2 × ChamQ Universal SYBR qPCR Master Mix (Vazyme Biotech Co., Ltd., Nanjing, China), 7.2 μL of sterile water, 0.4 μL of primer mix (10 μm each of forward and reverse primers). The designed primers for different genes amplification in qRT-PCR are listed in Table 1. Amplification procedure was as follows: 1 cycle of 30 s at 95°C, followed by 40 cycles of 5 s at 95°C and 15 s at 60°C, 1 cycle of 15 s at 95°C, 1 min at 60°C, 15 s at 95°C. The glyceraldehyde 3-phosphate dehydrogenase (GAPDH) gene was used as the internal control for normalization of gene expression level. Three replications were set for every sample. The LightCycler^®^ 480 (Roche) software version 1.5.1 was used for data analysis, and relative fold change of the targeted genes was calculated by using 2^-ΔΔCt^ algorithm [38].

**Table 1:** Primers sequences used for qRT-PCR amplification.

Gene	Primer	Sequence 5’ -3’
*SPS*	190259—F	CGCCTCAACGTCATCCC
	190259—R	TCCTCCGCCAGAAACCC
*SuSy*	383560—F	CTTGTTGCGTGTTTGCTTGC
	383560—R	GACCGACGGTGTCCTTGTTT
*CWIN*	71310—F	GAGCAGCCTGGACCTCACAC
	71310—R	GCGTAGAAGTTGCCGTAGTCG
*CeS*	332264—F	CAATATCAAAGAACAAGCTCACACC
	332264—R	ATCCAGGAGAAACCAAACCAGA

### Simple sequence repeats (SSRs) markers analysis

#### Leaf samples collection

Young leaf tissues were collected from six individual plants of each sugarcane clone at grand growth phases, rinsed with 80% ethanol, and stored at -80 ºC prior to DNA extraction.

#### DNA extraction and purification by SDS method

The SDS method (Xia et al., 2019) was used for DNA extraction with some modifications. A total of 2 g sugarcane leaf samples were ground to powder with 0.05 g PVP in liquid nitrogen by mortar and pestle. The powder was transferred into the liquid nitrogen cold microcentrifuge tubes (2 mL). Then 1.3 mL of SDS extraction buffer (pre-heated in 65°C) was added into the microcentrifuge tubes and mixed gently. The microcentrifuge tubes along with DNA and buffer were heated in water bath at 65°C for 60 min. Next the microcentrifuge tubes were taken out from water bath and 300 μL of 5M KAc were added, mixed gently, and put in ice bath for 15 min. Then the microcentrifuge tubes were centrifuged at 12,000 rpm, 4°C for 10 min, and the supernatant was transferred into a clean microcentrifuge tube (2 mL). Then equal volume of phenol-chloroform-isoamyl alcohol (25-24-1) was added to the homogenate and kept in ventilation hood for 1–2 min. After this the microcentrifuge tubes were centrifuged at 12,000 rpm, 4°C for 7 min to separate the liquid layers. The supernatant or upper phase was transferred to a clean microcentrifuge tube (1.5 mL), and repeated, then equal volume of phenol-chloroform-isoamyl alcohol (25-24-1) was added into the homogenate and kept in ventilation hood for 1–2 min. After this the microcentrifuge tubes were centrifuged at 12,000 rpm, 4°C for 7 min to separate the liquid layers and clear supernatant was obtained. Then the upper aqueous DNA containing phase was transferred to a fresh microcentrifuge tube (1.5 mL), 2 μL RNase was added, mixed well by gentle inverse mixing and incubated at 37°C for 30 min. Next equal volume of chloroform was added, vortexed for 1 min, and centrifuged at 12,000 rpm, 4°C for 7 min. The supernatant was transferred to clean microcentrifuge tubes (1.5 mL), equal volume of 80% ethanol (pre-cold at -20°C) and 1/10 vol. of 3 M sodium acetate (pH5.2) were added, mixed well and stored at -20ºC for 1 h. After this the tubes were centrifuged in 10,000 rpm, at 4°C for 10 min and the supernatant was discarded. 1 mL of 70% ethanol was added to wash the DNA and repeated two times. Then the DNA pellet was allowed to air dry for 12 min under clean bench until the ethanol evaporated out (not completely dried). 50 μL ddH_2_O were added to dissolve the DNA pellet and 1 μL of extracted DNA was taken to measure the OD at 260 nm and 280 nm by using Implen Nano Photometer^®^ P- Class (P300) for DNA amount determination. Finally, the DNA was stored at -20°C for PCR amplification.

#### DNA electrophoresis quantification

For electrophoresis, 2% agarose gel was prepared in 1× TAE buffer, and 4 μL DNA products together with loading dye were filled in the gel wells. The TAE buffer (1×) was used as running buffer in gel tank for 40 min electrophoresis at 120 V. The bands of extracted DNA quality were checked by using Gel Documentation System (Bio-Rad, USA).

#### PCR amplification

The PCR amplification was carried out in the TF professional basic thermocycler (Biometra, Germany) by using TSINGKE Biological Technology (www.tsingke.net) PCR primers (Table 2). The PCR mixture ingredients were 2 × TSINGKE (China) master mixes (blue), 2× (10 μM) forward, reverse primers, template DNA (~10 ng/μL) and ddH_2_O water. The PCR reaction was performed in a total volume of 20 μL, comprising of 10 μL master, mix, 1 μL each primer, 1 μL template DNA, and 7 μL ddH_2_O water. For 35 cycles of gradient PCR, temperature and time conditions were set as initial lid temperature 94°C (5 min), melting temperature 94ºC (30 s), annealing temperature 56–62ºC (30 s), extension temperature 72 ºC (60 s), and final holding temperature 72ºC (5 min).

**Table 2:** Primers sequences for PCR in SSR analysis.

Primers	sequences 3’-5’
SCB181	F: GGC GGC TGC TTC TGG GTT TGT
	R: GGA AGC CGA GGA GCA CGA GGAT
EST2–20	F: ATA AGA TCC GTG GTA GGG TAA
	R: AGG GAC GAA GGG AGT GC
SCB279	F: AGA GGG AGG ACA ACA ACA GG
	R: CTC CAG TCC CAG CAT AAA GAT
SMC1604SA	F: AGG GAA AAG GTA GCC TTG G
	R: TTC CAA CAG ACT TGG GTG G
SMC336BS	F: ATT CTA GTG CCA ATC CAT CTC A
	R: CAT GCC AAC TTC CAA ACA GAC

#### Polyacrylamide gel electrophoresis detection (DNA-PAGE)

The PCR amplification product was detected by PAGE separation method in Bio-Rad apparatus.

For preparation of 12% PAGE gel the volume of ingredients used were (29:1) 30% acrylamide (4.8 mL), ddH_2_O (4.8 mL), 5× TBE (2.4 mL), 10% APS (200 μL), and TEMED (10 μL). The gel was prepared in the falcon tube and TEMED was added at the last, because it immediately starts to react with APS and catalyzes the polymerization of acrylamide, so as a consequence the following mixing and casting steps have to be done as fast as possible.

The prepared gel solution was poured in the plates assembled with spacers and the comb was inserted immediately ensuring no air bubbles were trapped in the gel or near the wells. The gel was put for 2 h to set at room temperature. After the gel was ready, the glass plates were adjusted in the buffer tank, which was filled with 1× TBE running buffer up to the required level and the comb was removed. By using micropipette, 4 μL amplified DNA with 2 μL loading dye was filled in the gel well, and the electrodes were connected to a power pack, turning on the power to begin the electrophoresis for 3 h at 120 V.

After completion of the electrophoresis, the glass plates were taken out from the buffer tank, and both sides of the glass plate were rinsed with tap water. The gel was peeled off, and washed twice with double distilled water, then immersed in 0.1% AgNO_3_ solution, and shaken for 10 min. Again, it was rinsed twice with double distilled water for 12 s each time, and then submerged in 1.2% NaOH + 0.4% formaldehyde solution for development. Experimental results were analyzed in a gel documentation system (Bio-Rad, USA).

#### Statistical analysis of data

Statistical analyses of variance in expression of traits were done to know the significant difference of morphological, agronomical and physiological characteristics between the genotypes. Least significant difference (LSD) test at p < 0.05, < 0.01 was applied to find the differences among the means by using Statistix 8.1 software. Bar chart, stacked plot and line graph analyses were performed in Microsoft Excel 2016.

## Results

### Morphological and agronomical traits

#### Performance at germination stage

**Fig 1:**
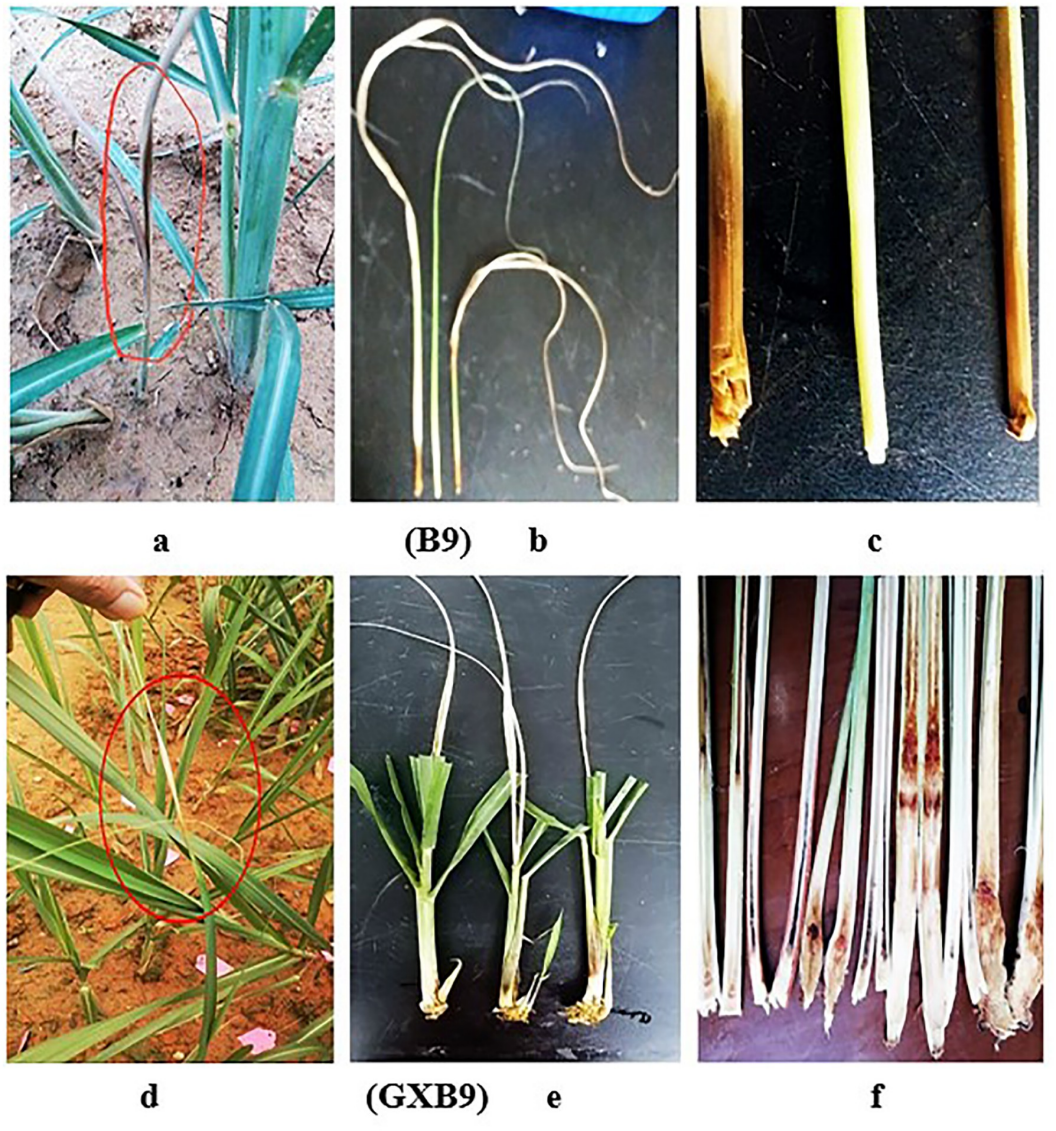
Heart-leaf withered plants occurred in B9 (a, b, c) and GXB9 (d, e, f) at late germination stage.

Throughout the regular observation at germination stage, it was noticed that the first two buds of B9 sprouted 10 days after planting, while the first 3 buds of GXB9 sprouted 12 days after planting. The emergence rate was higher in B9 than GXB9 (Table 3). In general appearance the seedlings were looking better in B9 than GXB9. At the last of germination stage, it was noticed that there were some heart-leaf withered plants, which were caused by borer damage, and the situation was severer in GXB9 than B9 (Fig 1).

**Table 3:** Comparison of agro-morphological traits between GXB9 and B9.

Traits	Stages	GXB9	B9
Emergence rate (%)	Germination	61±4.3	63±3.8**
Tillering rate (%)	Tillering	60±3.7	62±4.2**
Number of internodes	Maturity	25±2.3	27±3.8**
Number of green leaves	Maturity	10±1.5^ns^	11±2.2^ns^
Leaf length (cm)	Maturity	134.2±7.4	139.4±8.6**
Leaf width (cm)	Maturity	5±1.1^ns^	6±1.3^ns^
Internode length (cm)	Maturity	14.5±3.4	15.9±4.3*
Bud height (mm)	Maturity	12.3±2.5^ns^	10.5±3.4^ns^
Bud width (mm)	Maturity	9.4±3.5*	8.8±2.7

ns = non-significant,

* = significant at P≤0.05

** = highly significant at P≤0.01,

± values represent standard error among replications.

#### Performance at tillering stage

**Fig 2:**
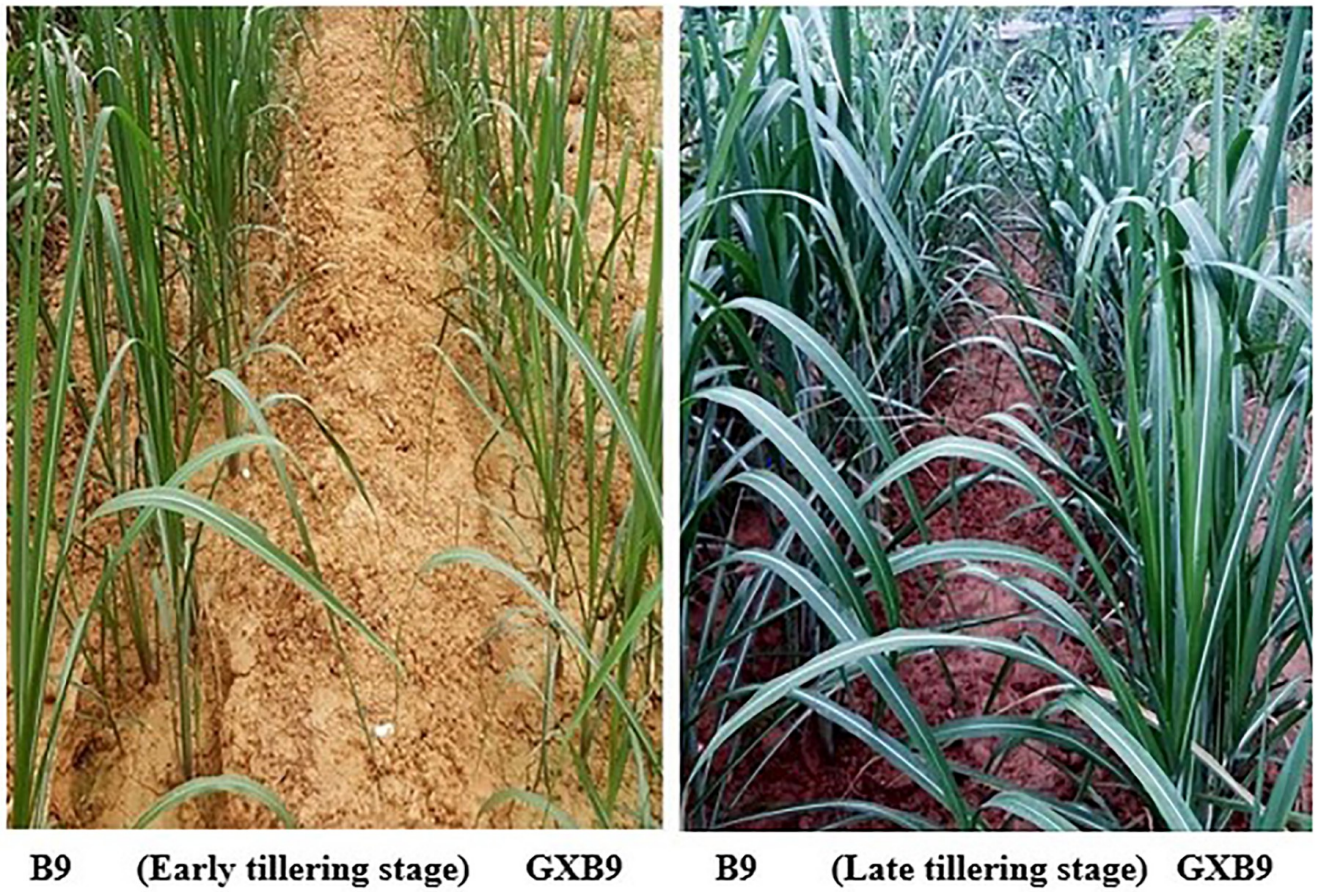
Plant growth status of GXB9 and B9 at early and later tillering stages.

At tillering stage, the plants growth status of B9 appeared better in than GXB9 (Fig 2), and B9 showed significantly higher plant height, stem diameter and leaf area than GXB9 (Table 4). The tillering rate was also higher in B9 than GXB9 (Table 3).

**Table 4:** Plant height, stem diameter and leaf area in GXB9 and B9.

Growth stage	Genotype	Plant height (cm)	Stem diameter (mm)	Leaf area (cm2)
Tillering stage	GXB9	59.84±0.42	7.36±2.0	94.65±0.25
	B9	70.56±0.17**	9.79±0.13**	109.40±0.06**
Grand growth stage	GXB9	291.3±0.65ns	17.25±0.81	378.47±0.68
	B9	296.5±0.37ns	19.42±0.22*	446.88±0.05**
Maturity stage	GXB9	314.2±0.25	23.65±0.18	503.25±0.32
	B9	316.5±0.26*	24.98±0.14*	923.60±0.09**

ns = non-significant,

* = significant at P≤0.05

** = highly significant at P≤0.01,

± values represent standard error among replications

#### Performance at grand growth stage

**Fig 3:**
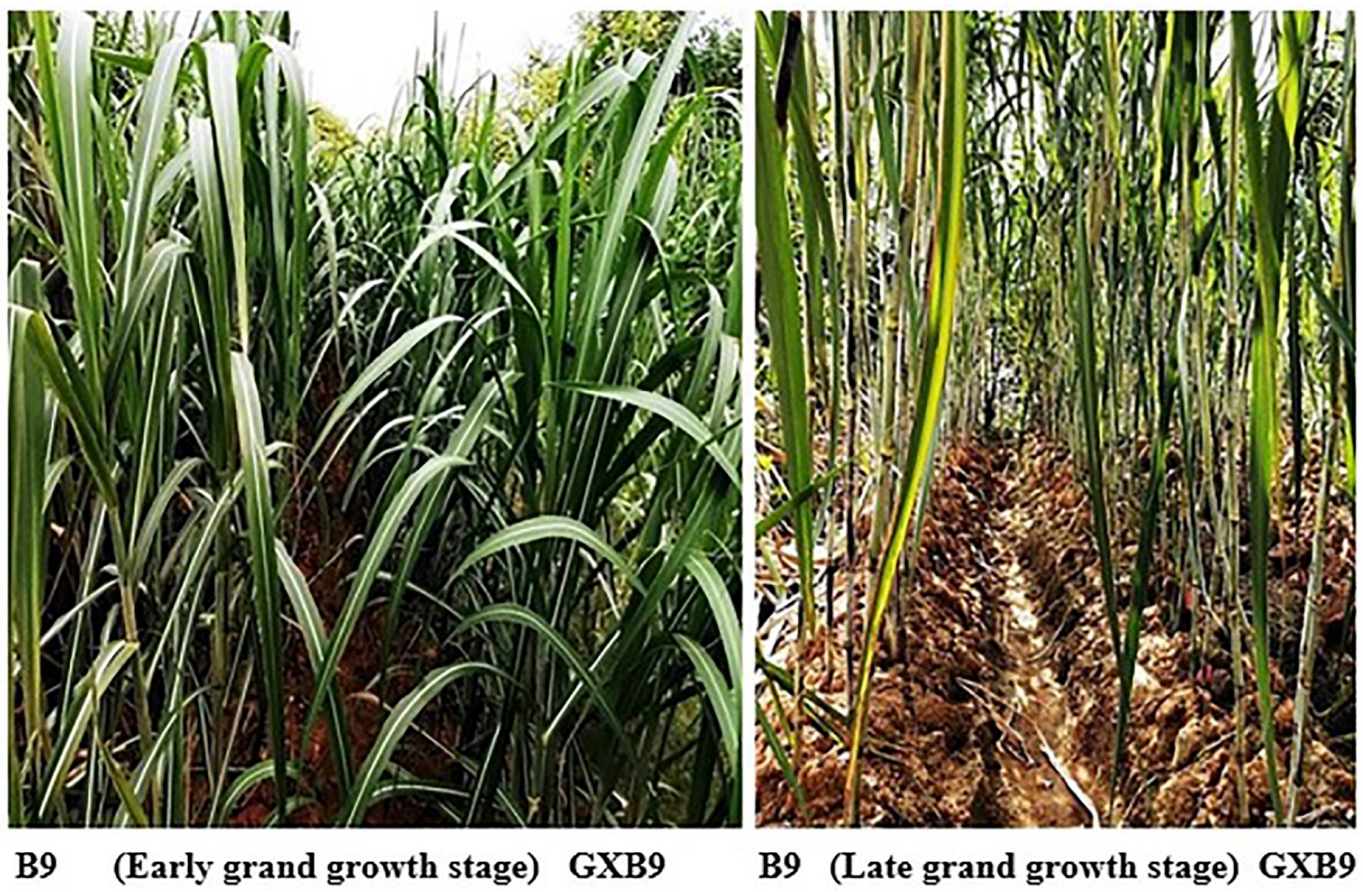
Plant growth status in GXB9 and B9 during grand growth stage.

At grand growth stage, both the sugarcane clones showed aggressive growth (Fig 3)., Plant height, stem diameter and leaf area were significantly higher in B9 than GXB9 at grand growth stage (Table 4).

#### Performance at maturity stage

**Fig 4:**
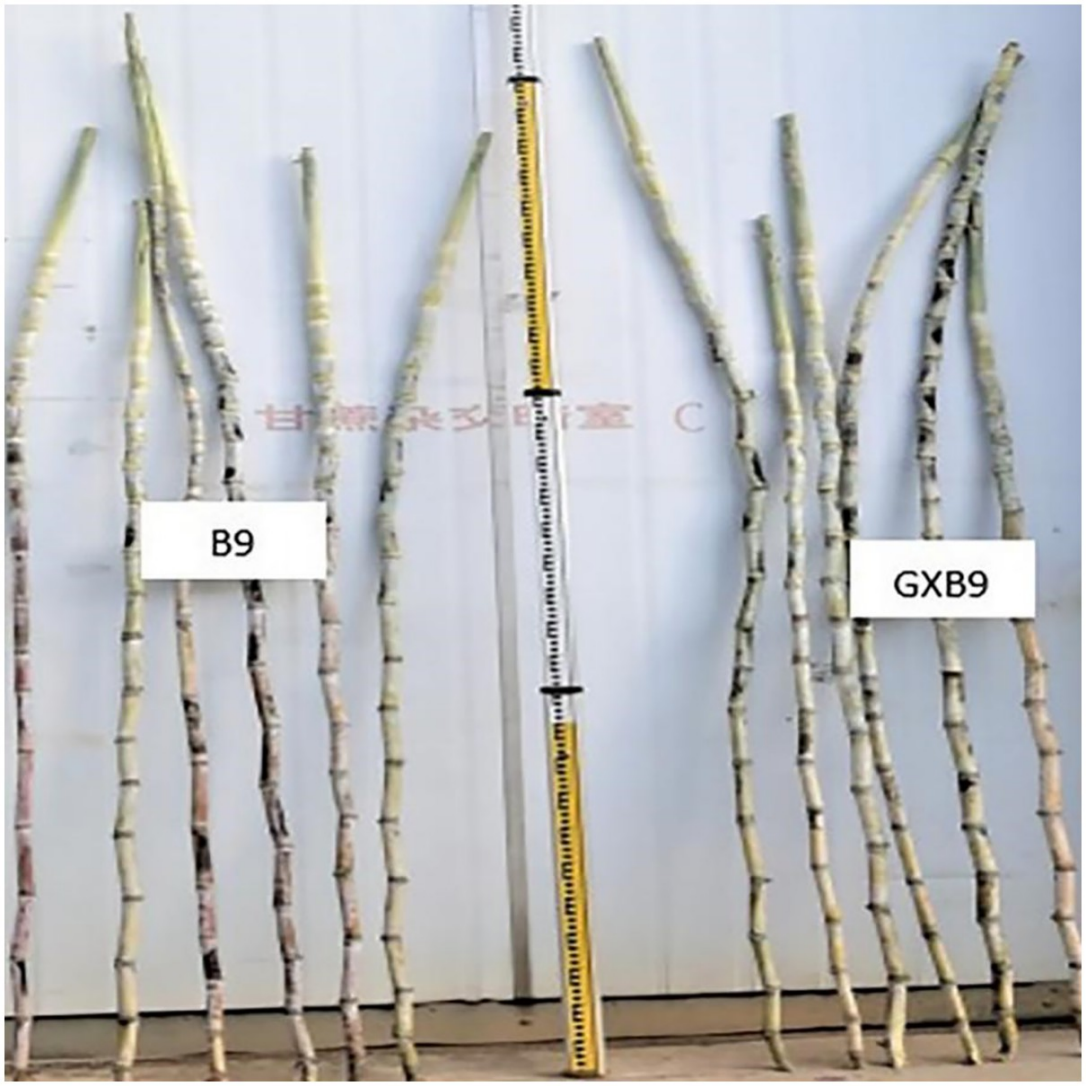
General view of the stalks in GXB9 and B9 at mature stage.

In general, the appearance of whole stalk looked similar between GXB9 and B9 (Fig 4). However, B9 still showed higher average number of green leaves per plant, leaf length and leaf width (Table 3), and significantly higher plant height and leaf area than GXB9 (Table 4)

#### Leaf morphological traits

**Fig 5:**
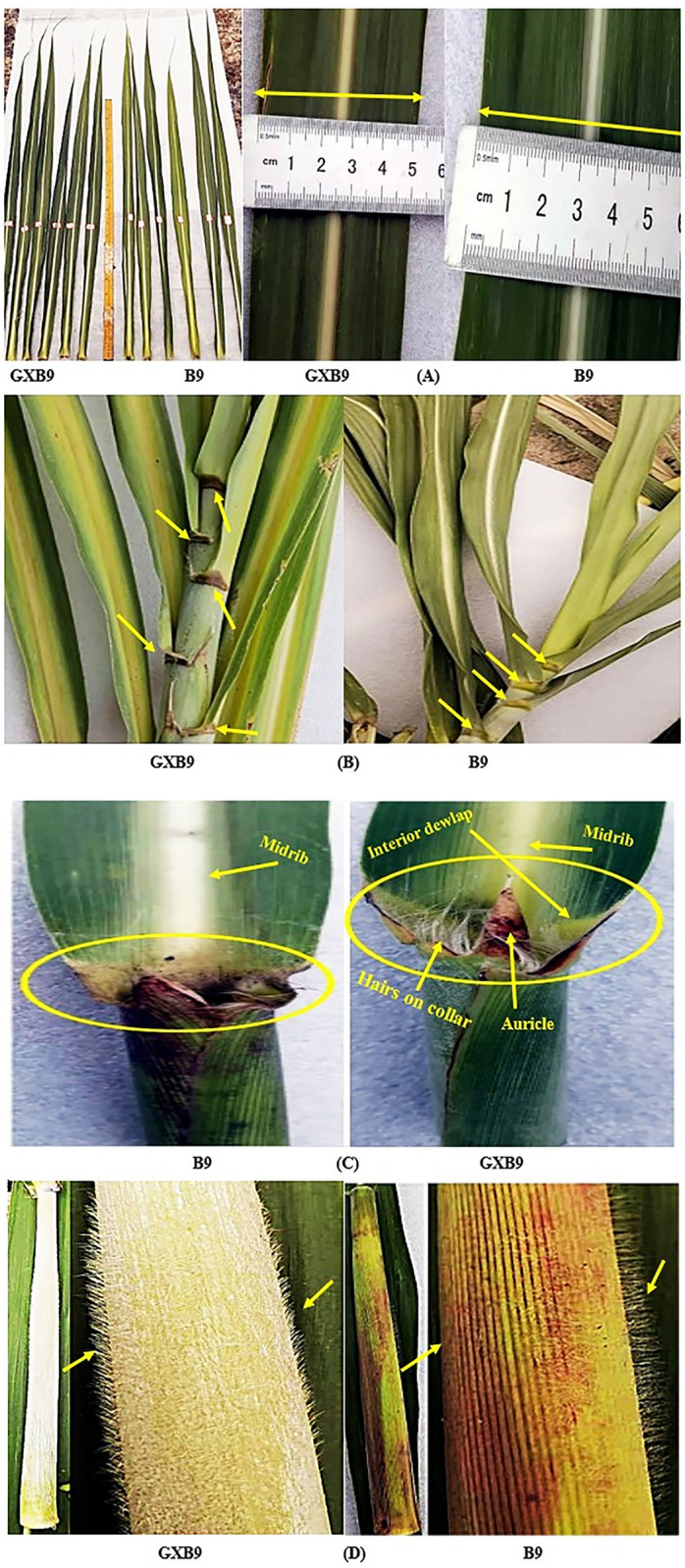
Leaf structures in GXB9 and B9. **A**, leaf length and width; **B**, ligule color; **C**, leaf auricle, collar hairs and dewlap; **D**, leaf sheath wax, spines and color.

The obtained data showed that B9 has significantly greater leaf length and leaf width than GXB9 at mature stage (Fig 5A, Table 3). The leaf dewlap color was dark chocolate on exterior side and light green on interior side in GXB9, while it was green in outside color but not visible on the interior side of leaf in B9 (Fig 5B). The auricle length was short in B9, but long and tapering in GXB9. GXB9 had more concentrated and erecter hair tufts on the collar of leaf compared to B9. The inverted deltoid shape dewlap on the inner side of leaf in GXB9 was clearly visible but absent in B9 (Fig 5C). The leaf sheath of GXB9 showed comparatively more wax, milky white in color, and thicker spines compared to B9 which was green in color with less spines (Fig 5D).

#### Internode length

**Fig 6:**
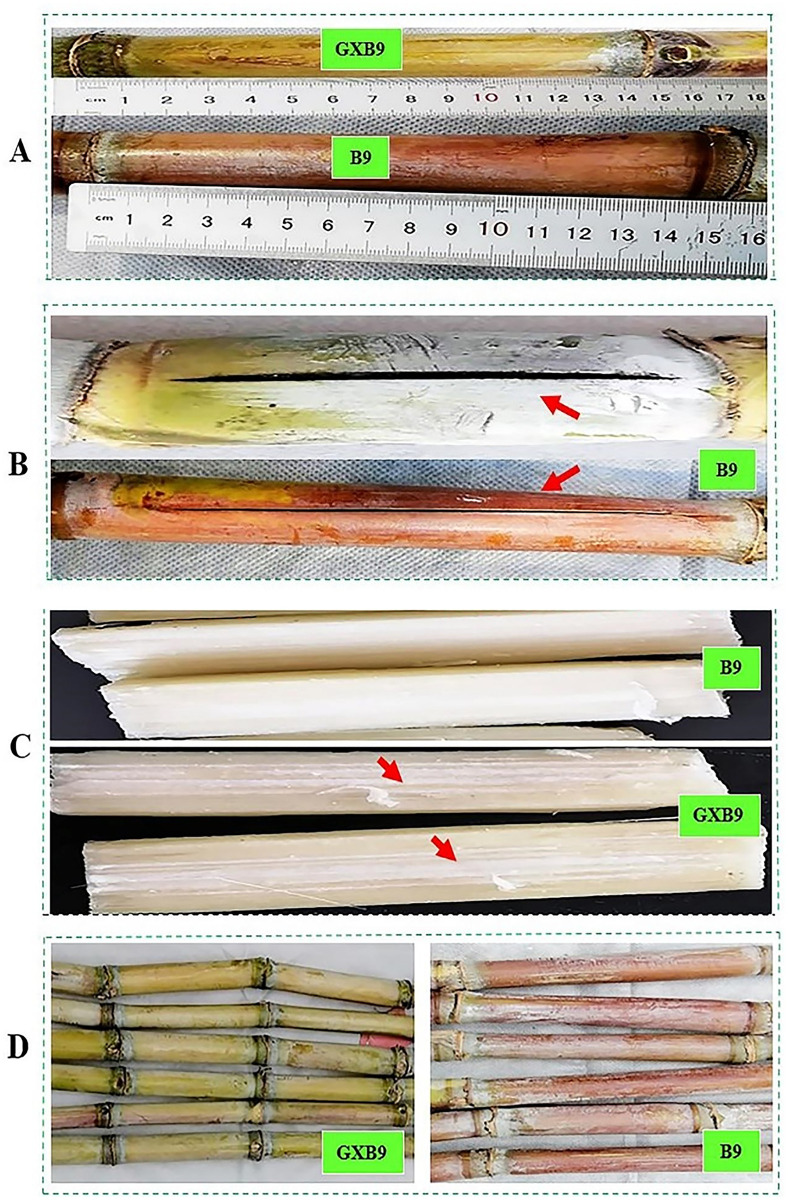
Internode characteristics in GXB9 and B9. **A**, internode length; **B**, growth crack; **C**, internal view of internode; **D**, internode color.

The measurement and comparison of internode length between GXB9 and B9 at mature stage displayed that mean length of internode was comparatively higher in B9 than GXB9 (Table 4). All the internodes were cylindrical in shape, but GXB9 mutant clone showed more zigzag alignment however less internode length than B9 (Figs 4 and 6A). Growth cracks were present in maturing and ripened internodes of B9 (Fig 6B). However, no cracks were found in the internode of GXB9. The longitudinal dissection of internodes at mature stage showed visible spongy pith lines in GXB9 mutant compared to B9 (Fig 6C). The internodes exposed to sunlight showed different colors between GXB9 and B9 throughout the course of growth. The internodes of GXB9 displayed a light green or greenish color while wine red or purple color was observed in the internodes of B9 mother clone (Fig 6D).

#### Bud characteristics

**Fig 7:**
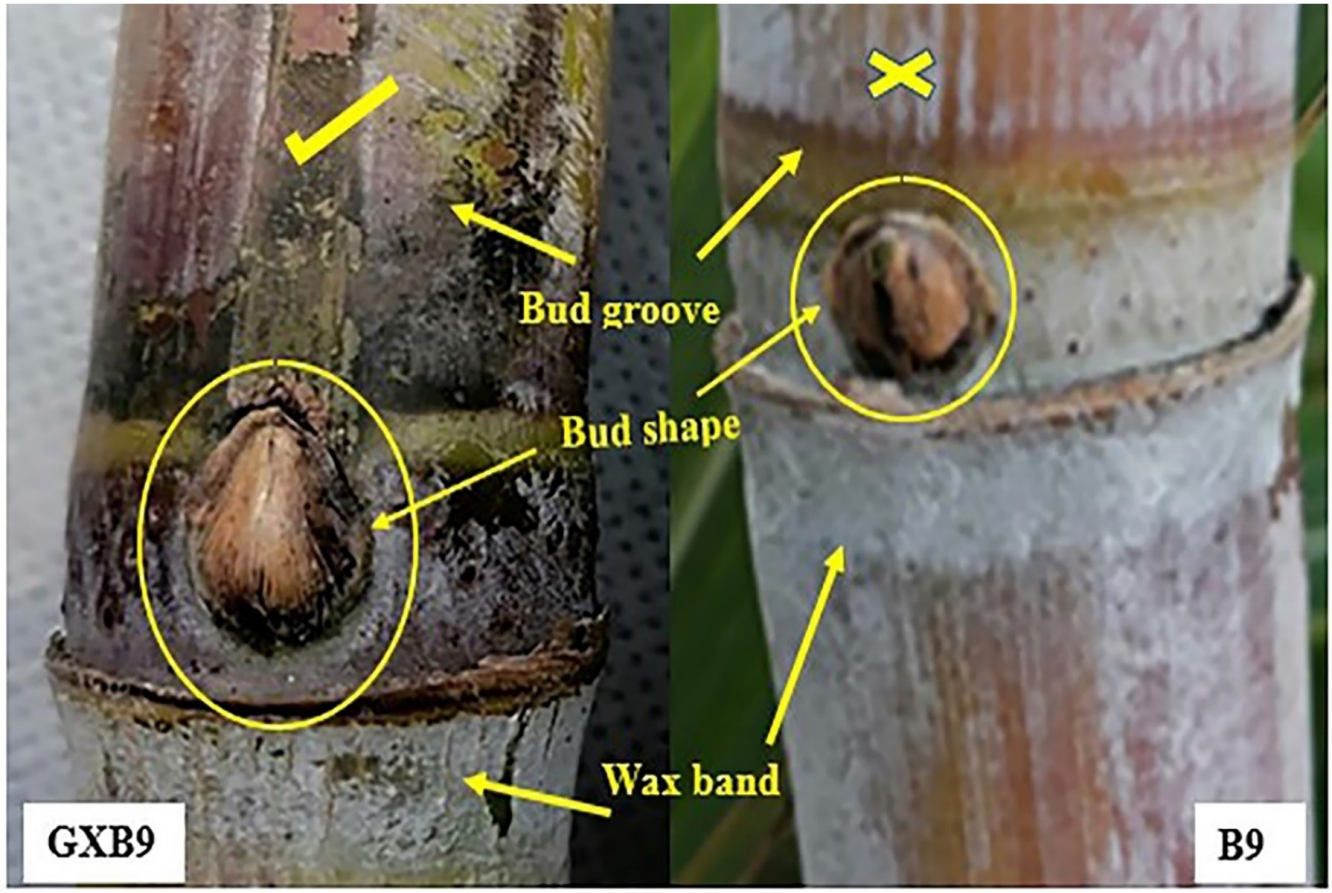
Comparison of bud characteristics between GXB9 and B9 at mature stage.

The observation of buds showed visible variation between GXB9 and B9. In GXB9, the bud position was above the leaf scar, and there was obvious gap between the bud position and leaf scar, while in B9, the bud position was on the leaf scar and the tip did not cross the growth ring. The bud size in GXB9 was bigger and the tip crosses the growth ring, and the bud was ovate to triangular pointed in shape with deep and long eye furrow or bud groove, while the bud shape was round in B9 projected in space with no or very small bud groove. The bud dimensions like height and width recorded were higher in GXB9 than B9 (Fig 7, Table 3).

#### Physiological parameters

**Fig 8:**
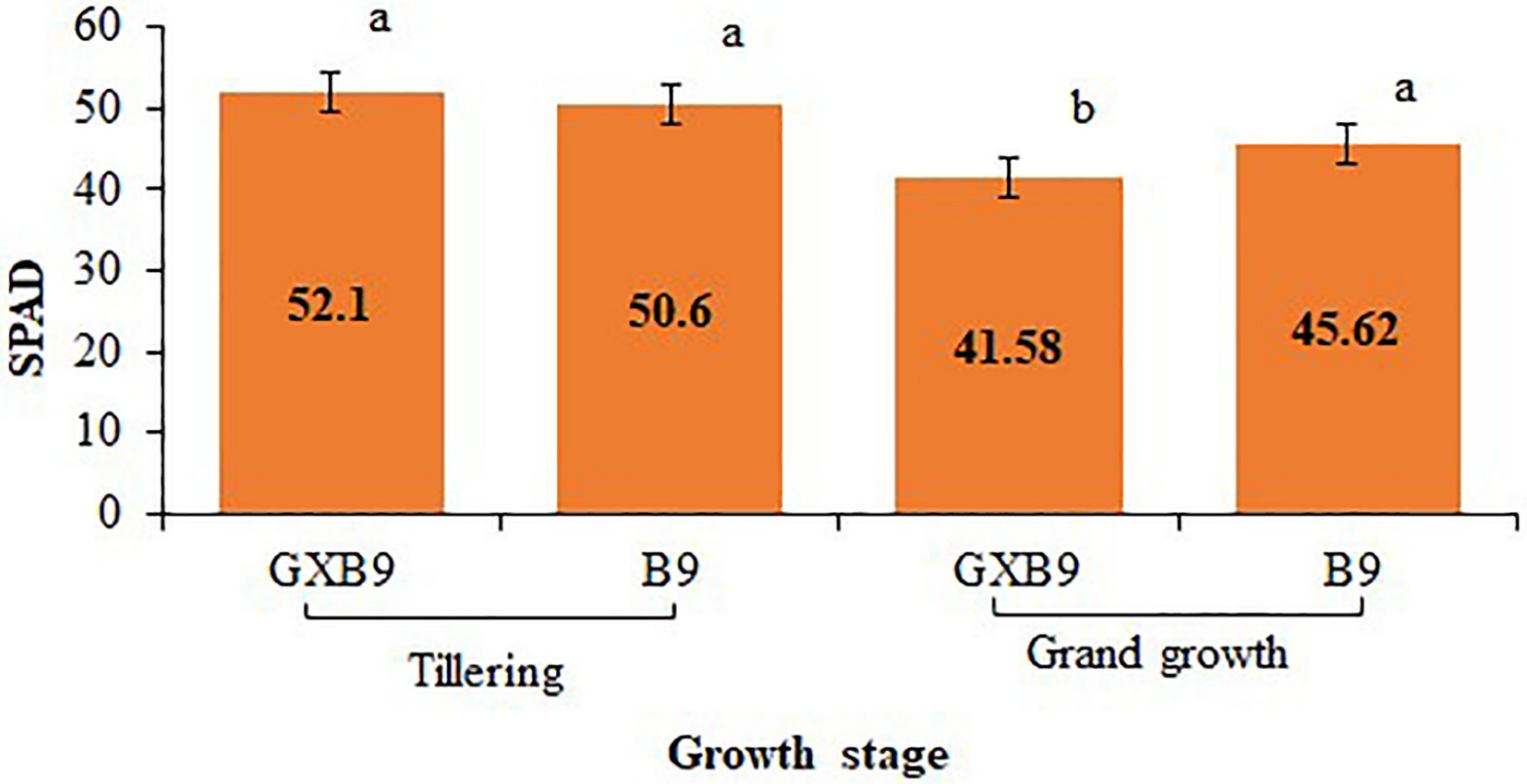
SPAD in GXB9 and B9. Values labeled with the same letters above columns are not significantly different at p ≤ 0.05.

*SPAD*. The leaf SPAD readings at the tillering stage were higher than that at grand growth stage in both varieties. There was no significant difference between GXB9 and B9 at tillering stage, however it was significantly higher in B9 than GXB9 at grand growth stage (Fig 8).

*Chlorophyll content and photosynthetic parameters*. Results showed the contents of chlorophyll a and b in leaves were higher in GXB9 than B9 (Table 5). For photosynthetic parameters including Pn, gs, Ci and Tr, GXB9 showed significantly higher value than B9 except for Ci (Table 5).

**Table 5:** Summary of photosynthetic components and chlorophyll evaluation.

Parameter	GXB9	B9
Net photosynthetic rate (Pn) (μmol m-2 s-1)	26.5±0.15*	23.71±0.01
Stomatal conductance (gs) (mol m-2 s-1)	0.24±0.005*	0.17±0.005b
Intercellular CO2 (Ci)(vpm)	113.33±0.27ns	114±0.001ns
Transpiration rate (Tr) (mmol m-2 s-1)	10.81±0.04**	8.63±0.005
Chlorophyll a (mg g-1)	2.88±0.9*	2.69±0.01
Chlorophyll b (mg g-1)	0.78±0.005*	0.73±0.02

ns = non-significant,

* = significant at P≤0.05

** = highly significant at P≤0.01,

± values represent standard error among replications.

**Fig 9:**
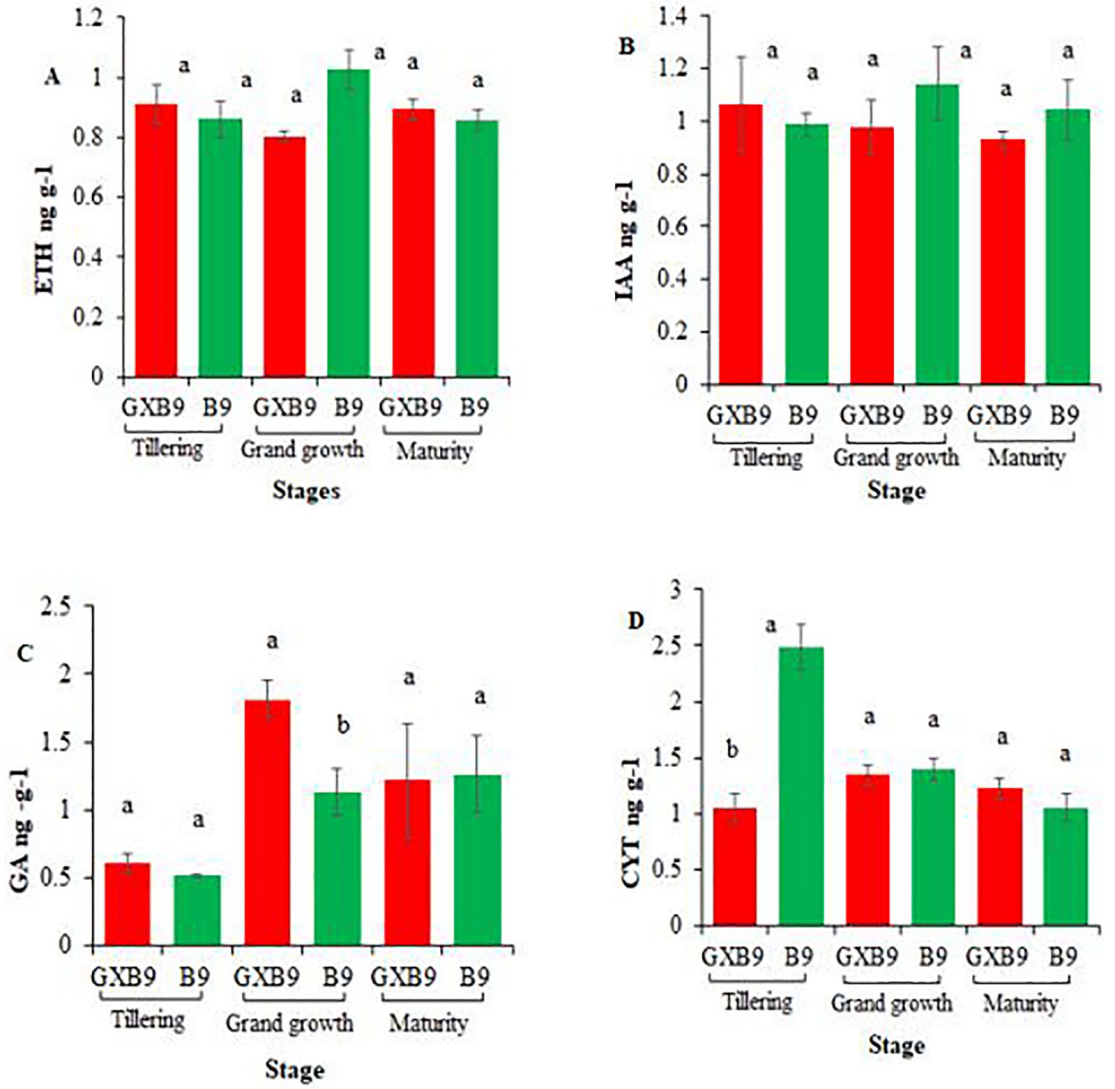
Endogenous phytohormone concentrations in GXB9 and B9 at different growth stages (A) Ethylene (ETH), (B) Indole acetic acid (IAA), (C) Gibberellic acid (GA), (D) Cytokinin (CYT). Different small letters above the column indicate significant difference at p ≤ 0.05.

*Endogenous hormones*. The endogenous hormones quantification at tillering, grand growth and maturity stages presented variation between GXB9 and B9. The concentration of endogenous hormones ETH and IAA showed no significant variation between the two genotypes at all the growth stages (Fig 9A and 9B). The concentration of GA was recorded with no significant difference between the two clones at tillering stage and maturity stage, however, it was significantly higher at grand growth stage in GXB9 than B9 (Fig 9C). The cytokinin (CYT) quantity was significantly higher in B9 than GXB9 at tillering stage, but not significantly different at later stages (Fig 9D).

*Relative expression of selected genes analyzed with qRT- PCR*. The relative expressions of selected genes in the maturing and immature internodes were analyzed at maturity stage. The results showed that genes encoding enzymes such as SPS (SPS5), SuSy, CWIN and CeS were expressed in the internodal tissues of both sugarcane clones. However, all these genes were upregulated in GXB9 while downregulated in B9 (Table 6). The upregulated genes presented variation in fold change (FC) between the immature and maturing internodes of GXB9 (Table 7). The expression level of SPS5 was significantly higher in the maturing internodes than the immature internodes, while those of SuSy, CWIN and CeS presented significantly higher relative expression in the immature internodes than the maturing internodes.

**Table 6:** Relative gene regulation in GXB9 mutant and B9 mother genotype.

Genes	Internodes	Fold change	Regulation	Fold change	Regulation
		GXB9	GXB9	B9	B9
*SPS*	Immature	0.54	↓	0.87	↓
	Mature	1.04	↑	0.91	↓
*CeS*	Immature	4.02	↑	0.23	↓
	Mature	3.50	↑	0.26	↓
*CWIN*	Immature	7.88	↑	0.11	↓
	Mature	0.27	↓	0.83	↓
*SuSy*	Immature	3.89	↑	0.22	↓
	Mature	1.51	↑	0.61	↓

Genes with fold change (FC) value ≥ 1 were upregulated and vice versa

**Table 7:** Genes relative expression in immature and mature internodes of GXB9.

Selected genes	Immature internodes	Mature internodes
*Sucrose phosphate synthase* (*SPS*)	0.54±0.01	1.04±0.03**
*Sucrose synthase* (*SuSy*)	3.89±0.11**	1.51±0.08
*Cell wall invertase* (*CWIN*)	7.88±0.09**	0.27±0.02
*Cellulose synthase* (*CeS*)	4.02±0.06*	3.50±0.07

* = significant at P≤0.05

** = highly significant at P≤0.01,

± values represent standard error among replications.

*Cane yield and its components*. The theoretical cane yield and its components such as single stalk weight and millable stalks per hectare were investigated. The results (Table 8) showed that the single stalk weight was significantly lower in GXB9 mutant than B9 mother clone, but the millable stalks per hectare was higher in GXB9 than B9. The theoretical cane yield was significantly higher in B9 than GXB9.

**Table 8:** Theoretical cane yield and its components in GXB9 and B9.

Components	GXB9	B9
Single stalk weight (kg)	1.25±0.07ns	1.55±0.09ns
Millable stalks per hectare	63333±372 **	58333±462
Theoretical yield per hectare (ton/ha)	79.06±5.8	90.48±8.5**

ns = non-significant,

* = significant at P≤0.05

** = highly significant at P≤0.01,

± values within the columns represent standard error among replications.

Accordantly, GXB9 showed better cane juice quality than B9 as it had significantly higher juice rate, juice gravity purity, juice brix, and juice sucrose content compared to B9 (Table 9).

**Table 9:** Cane juice quality traits in GXB9 and B9.

Trait	GXB9	B9
Juice Brix (Bxo)	21.33±3.3*	19.90±3.6
Juice sucrose (%)	19.94±2.8*	18.41±3.1
Juice yield (%)	60.78±4.6**	58.15±5.8
Juice gravity purity (%)	92.94±4.1**	91.66±7.2

* = significant at P≤0.05

** = highly significant at P≤0.01,

± values within the columns represent standard error among replications.

**Fig 10:**
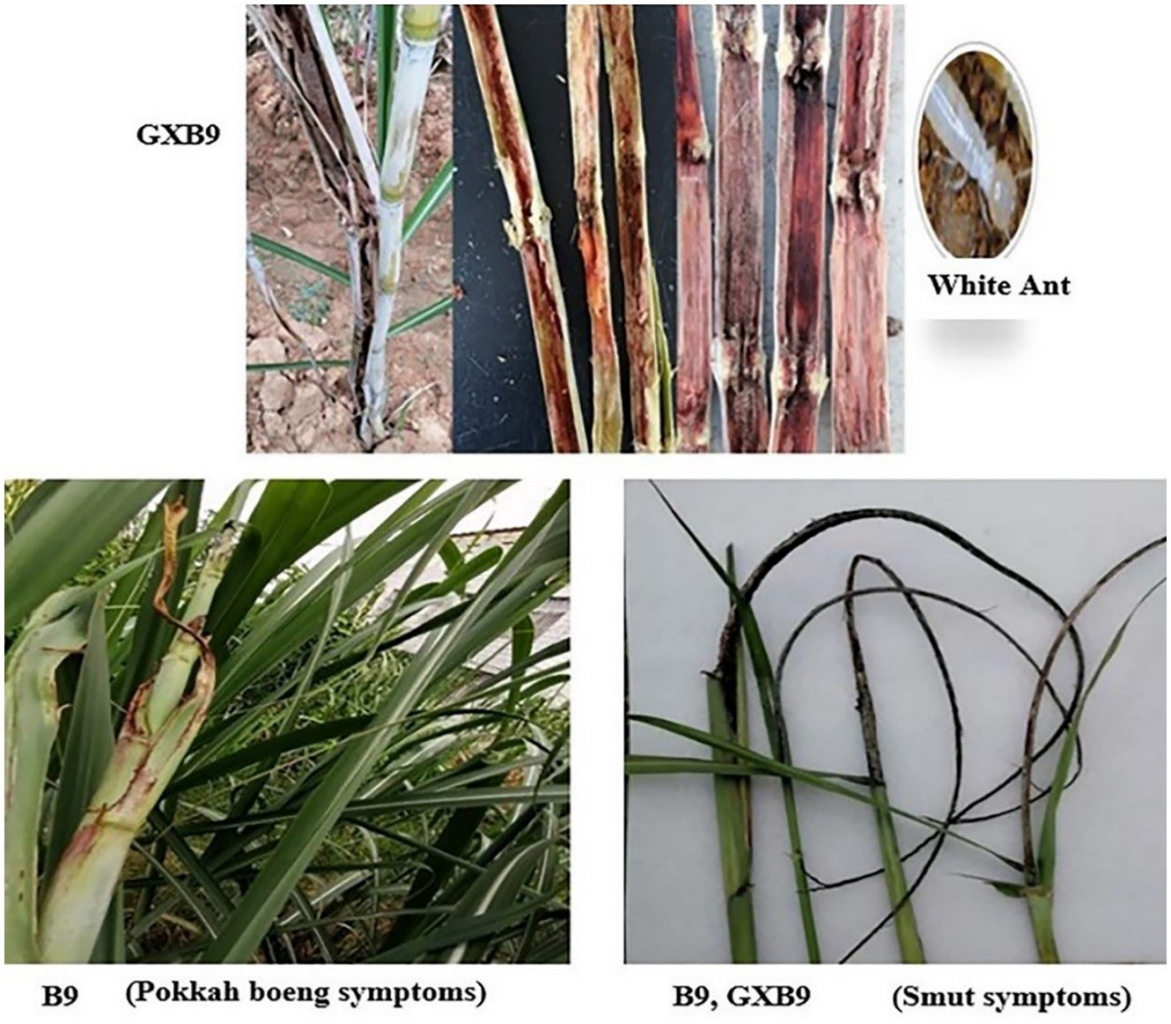
Occurrences of some diseases and pests attack in sugarcane plants of GXB9 and B9.

*Diseases and pest observation*. During the crop growth, occurrences of different diseases and pests attack in both clones were observed. It seems that smut occurred more in GXB9 than B9, but the incidence was at low level in both genotypes (Fig 10). The pokkah boeng disease symptom was observed only in B9 at low level at maturity stage (Fig 10). Besides borer attack, it was also found that the stalk of GXB9 was seriously attacked by white ants at the sugar accumulation and maturing stage (Fig 10), so high attention should be paid to control white ant for this genotype.

### SSR analysis

#### DNA extraction

**Fig 11:**
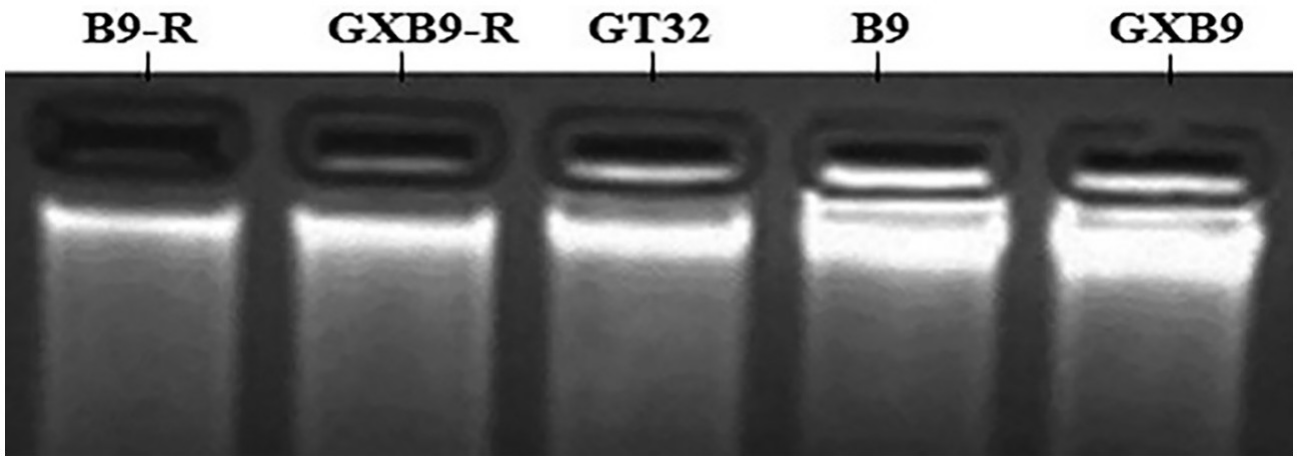
Results of genomic DNA detection with gel electrophoresis.

The DNA extracted by SDS method was detected at 260 nm and 280 nm by Implen Nano Photometer^®^ P- Class (P 300). The DNA quality was also checked by electrophoresis with 2% agarose gel in 1× TAE buffer (Fig 11). The results showed that the quality of extracted DNA was good enough for further analysis.

#### Polyacrylamide gel electrophoresis detection by PAGE analysis

**Fig 12:**
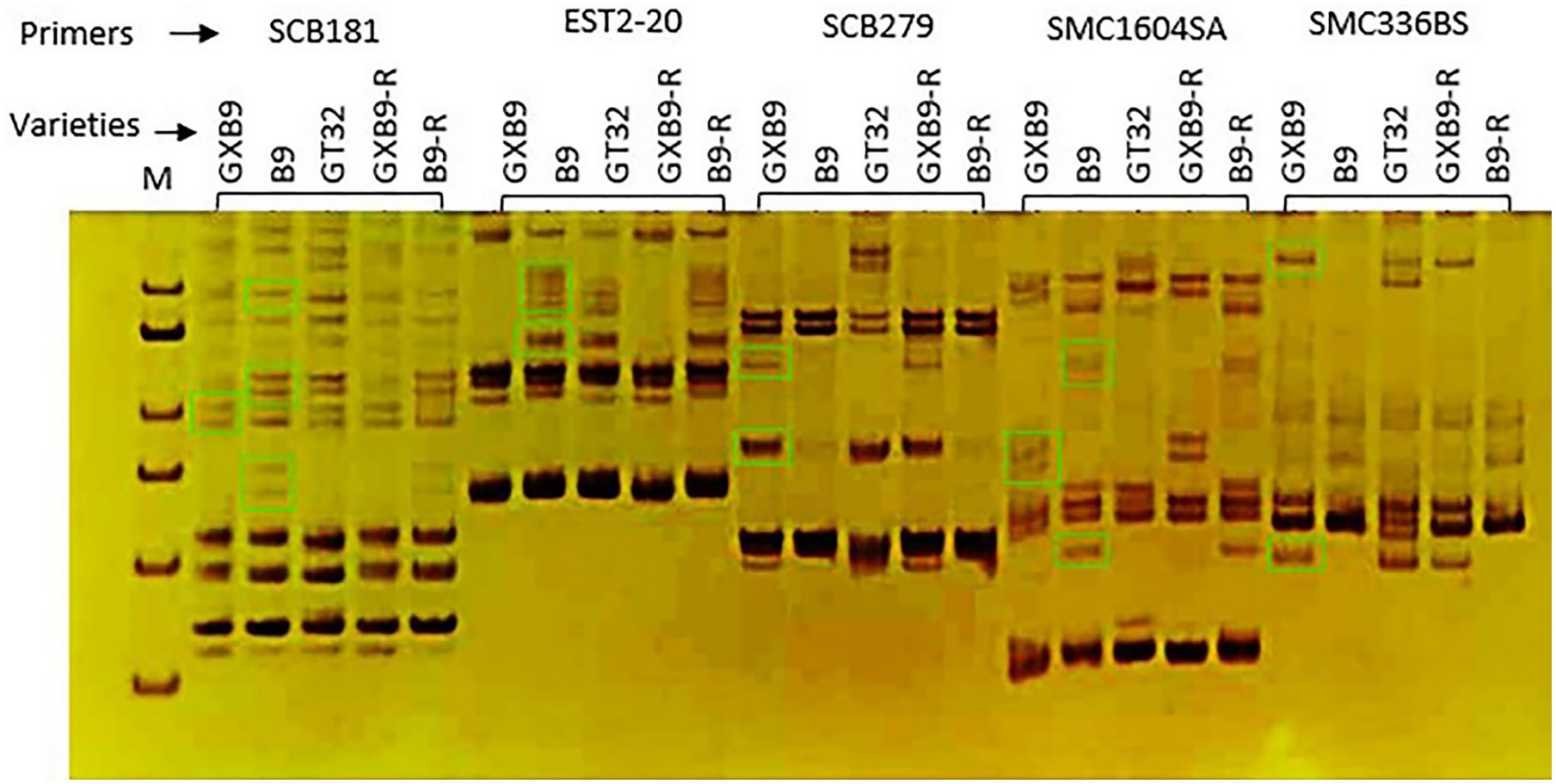
SSR band profiles for three different sugarcane clones analyzed by PAGE. From left to right, every five lanes corresponded to one primer and five templates. The template sequences were GXB9 plant cane (GXB9), B9 plant cane (B9), GT32 plant cane (GT32), GXB9 ratoon cane (GXB9-R), B9 ratoon cane (B9-R) while the primers were SCB181, EST2-20, SCB279, SMC1604, SMC336BS, respectively. M, molecular marker.

The results of PAGE analysis (Fig 12) showed that both plant and ratoon canes of GXB9 (GXB9 and GXB9-R) had the same SSR marker band profile, and so did those of B9 (B9 and B9-R), but the profiles of GXB9 had different bands from those of B9 including B9-R, and GT32. The SSR marker band profile of GT32 was also different from those of B9, reflecting that GXB9, B9 and GT32 are three different sugarcane genotypes.

## Discussion

Sugarcane is the most important crop globally for sugar and bio-ethanol production. Agronomical, morphological and physiological traits of sugarcane are very significant markers for identification of a crop genotype and new sources of variability. Growth, yield and yield components are important traits to evaluate cultivars of sugarcane. Mutagenesis is a process through which the genetic information of an individual is changed in a long-lasting manner, resulting in a mutation. Natural mutagenesis can produce a new organism that may be beneficial, harmful, or has no effects on other organisms. Spontaneous mutations are because of errors in natural biological practices, while induced mutations are because of agents in the environment that originate changes in DNA structure [38]. However, mutation brings changes in the traits of a genotype within a population [39]. Spontaneously occurred mutations furnish raw materials for natural selection (NS) and evolution in all organisms [40].

The focus of present exploration was to investigate a high sucrose content sugarcane mutant GXB9, on morphological, agronomical, physiological and molecular levels in comparison to its low sucrose content sugarcane mother clone B9 to find the variation in qualitative and quantitative traits, as well as expression of genes associated with sucrose metabolism in GXB9.

The general common observations recorded for both genotypes during all the growth phrases were medium germination rate, erect plant and resistant to wind pressure, green leaves with semi dropping projection and easy to fall off, light green (covered) cylindrical internodes with zigzag alignment to some extent. About forty-two qualitative and quantitative characteristics including morphological, agronomical, physiological and molecular traits were measured and analyzed in this study, among which most have shown significant variation in GXB9 in comparison with B9.

It was found that GXB9 had significantly lower plant height, stalk diameter, leaf area, leaf length and leaf width than B9. It is thought that these variation in the traits of GXB9 are due to mutation, and previously it has been reported that spontaneous mutation causes morphological variation in sugarcane [41].

Based on the results of the present study, GXB9 has longer auricles with tapering tip, thick hairs tuft on the leaf collar, a clearly visible light green anterior dewlap with dark chocolate color at posterior, and slightly yellowish midrib in contrast to B9 which has shorter auricle, less hairs on collar, no visible anterior dewlap mark but visible posterior dewlap with light green color and whitish midrib. The leaf sheath of GXB9 is milky whitish in color with thick waxy powder and has very concentrated spines in comparison to B9 whose leaf sheath is green in color, very sparse wax powder and smaller number of spines. These variations in GXB9 in comparison to B9 under the same environmental conditions indicate that mutation has occurred in GXB9. It has been described recently that sugarcane varieties having genetic variability showed variation in morphological traits [42–44].

Variations in size, shape and other characteristics of the bud provide means to distinguish varieties. The results of current study indicated that GXB9 has difference in bud shape, size, position, cushion, extension, and bud groove in contrast to B9. The bud of GXB9 has bigger size, ovate to triangular in shape, with tip that crosses the growth ring, obvious gap between bud position and leaf scar, deep bud groove in contrast to B9, in which shape of bud is round and tip projecting in space, bud present at the leaf scar and smaller in size. These variations again support the perception of mutation in GXB9 clone, because any kind of mutation causes variation in traits among the clones of a population [45–47].

GXB9 has comparatively shorter length of internodes but very visible internal spongy pith lines. The pith decreases yield but causes increase in sucrose content of sugarcane [48]. The stalks or internodes of both varieties exposed continuously to sun light during the course of growth displayed difference in color. The internodes in GXB9 retained light green color, while those in B9 showed wine red color. Further, growth cracks or ivory marks were only observed in the internodes of B9.

Leaf area determines the amount of incident PAR intercepted by the crop canopy and ultimately increase dry matter production and the transformation of solar energy into chemical energy by photosynthesis which is directly related to yield [49, 50]. Leaf area is an important trait associated with growth rate and yield of plants. So, improved photosynthetic rate combined with large leaf area maybe the way forward to increasing photosynthesis and for future efficiency of crops [51]. In this study GXB9 showed less leaf area than B9, which may be contributing to more light receiving, increased photosynthesis and enhanced biomass production in B9 [52].

Leaf chlorophyll concentration is an important parameter that is commonly measured as an indicator of chloroplast development, photosynthetic capability, leaf nitrogen level or general plant health. Measurements with the SPAD-502 meter, calculate relative SPAD values which are proportional to the amount of chlorophyll existing in leaf [53]. Chlorophyll content is almost proportional to plant N content. Chlorophyll is a green-reflective substance considered as indicator of photosynthetic capacity, productivity, and stress levels [54]. In this study, SPAD readings in the leaves of GXB9 and B9 showed no significant difference at tillering stage, however, GXB9 showed significantly higher SPAD readings than B9 at grand growth stage. Moreover, Chlorophyll a, b concentrations did not show significant variation between GXB9 and B9, which indicated that both genotypes have similar chlorophyll content.

Photosynthesis is the foundation of cane sugar yield formation in sugarcane. It is known that around 90–95% of crop production is obtained from assimilated carbon. So, enhancement in crop photosynthetic ability is one of the most significant targets in genetic studies and crop breeding [55]. Pn denotes the value of plant photosynthesis, and measurement of Pn is of practical importance in defining the level of carbon fixation and endorsing the plant growth and development [56]. Pn is a highly significant index in assessing CO_2_ emission reduction. An accurate prophecy of Pn could be a convincing indication for analyzing and estimating carbon sequestration. The increase in Pn enhances cane sugar yield [57], and sucrose production is importantly correlated with Pn of leaf [58]. The results in current study indicated that GXB9 has significantly higher Pn than B9, which may be contributing to higher sucrose content in GXB9 and this view has also supported by literature [59–61].

Different kinds of phytohormones play their roles in various physiological activities of plant and five plant hormones, namely auxin, gibberellins, cytokinin, abscisic acid and ethylene are very important [62]. Cytokinin comprises a family of signaling molecules essential for regulating the growth and development of plants, acting both locally and at a distance [63]. Ethylene is considered a ripening hormone in plants which backs to increase the storage of sucrose in sugarcane [64]. GA_3_ is a scientifically well-known hormone [65, 66]. Diverse isoforms of GA have important roles in growth and development of plants chiefly leaf morphogenesis, floral development and fruit ripening [67]. The function of auxin has been widely reported in sugarcane and it plays a role in plant growth and development [68]. In the current study it was found that cytokinin quantification was significantly higher at tillering stage in B9 than GXB9, while gibberellin was recorded significantly higher at grand growth stage in GXB9 than B9. These results indicated that GXB9 and B9 have significant differences in some phytohormones at different growth stages.

Sugar production in sugarcane depends on cane yield and sucrose content in cane. Analysis of cane yield and quality traits in this study discovered that GXB9 had significantly lower single stalk weight, but significantly higher number of millable stalks per hectare than B9 mother clone, finally, B9 had significantly higher theoretical cane yield than GXB9. Sucrose content in cane is highly correlated with brix. The greater the value of brix, the higher is the sucrose content in cane. On the pitch of sugarcane, the term Commercial Cane Sugar (CCS), or sugar quality index, defines the maximum percentage of sugar from fresh sugarcane [69]. In this study, GXB9 had significantly higher juice rate, juice gravity purity, brix, sucrose in juice, and sucrose in cane than B9. These results strongly advocate the argument that variations in the traits of GXB9 in comparison to B9 are due to mutation. Previous literature reported that genetic manipulation, either induced or spontaneous beneficial mutation has contributed to agronomic traits of sugarcane [70–74]. GXB9 and B9 together should be a couple of good materials for studying the molecular mechanism of sugar accumulation in sugarcane as they have similar genetic background but significant difference in cane quality traits especially sucrose content in cane.

SPS is a noteworthy enzyme which has active influence to manufacture sucrose in diverse species of plants [75, 76]. In addition to sucrose synthesis, SPS is also linked to many valuable agronomic characteristics mainly plant height and improved yield [77, 78]. SuSy is actively functional in immature portions of sugarcane stalk [79, 80], and is negatively correlated with sucrose accumulation [81]. CWIN is the important enzyme in sucrose metabolism which catalyzes the irreversible hydrolysis of sucrose into hexoses. CeS fabricates polysaccharides named cellulose which is a chief constituent of plant cell wall. In the present study, the relative expressions of genes coding for SPS, SuSy, CWIN and CeS enzymes in qRT-PCR analysis were highly upregulated in the internodes of GXB9. CeS, SuSy and CWIN showed higher expression levels in the immature internodes than the maturing internodes in GXB9, which might promote the sugar availability for growth and development in the immature internodes of the high sucrose content mutant. These results are consistent with the reports of [82–84]. In the present study, higher expression of SPS is linked with higher quantity of sucrose in the mature internodes while lower level of SPS expression is connected with lower sucrose in the immature internodes, which is in agreement with the results reported by [85], but inconsistent to the results observed by [86, 87] who reported immature internodes have higher SPS activity than mature internodes.

SSR markers have been extensively used to explore the genetic diversity and population structure [21, 88], clone identity [89], genetic map [90], and genetic association [91] in sugarcane. Further, SSR markers are also applied to confirm the offspring in F1 population of sugarcane by breeders to identify true offspring and thus help to advance the sugarcane crossing programs [92]. The result obtained from the SSR marker analysis in the present study revealed the genetic variations among the sugarcane genotypes GXB9, B9 and GT32, which proved mutation in GXB9 from B9.

## Conclusion

In summary, the results of morphological, agronomic, physiological and molecular analyses confirmed the variations of GXB9 mutant from its B9 mother clone. In this study, morphological, agronomical, physiological and molecular characterization of GXB9 in comparison to B9 showed meaningful and significant variations which advocates to declare GXB9 as a mutant clone of B9. Although both clones have similar appearance but there were numerous significant variations in morphological, agronomical, physiological and molecular level, and the yield related traits such as net photosynthetic rate, brix, and sucrose content in cane were significantly higher in GXB9 than B9, reflecting GXB9 is a high sugar mutant generated from B9, a low sugar mother clone. GXB9 and B9 have similar genetic background but different sucrose content in cane so both together would be excellent materials to study the molecular mechanism of sugar accumulation in sugarcane. Also, the current finding could be an important reference for future sugarcane breeding, particularly in sucrose content improvement.

## Supporting information

S1 Data (XLSX)

S1 Raw image (JPG)

S2 Raw image (JPG)
