# Supplementary figures and images for: Morphological, agronomical, physiological and molecular characterization of a high sugar mutant of sugarcane in comparison to mother variety
Source: PLoS One. 2022 Mar 10;17(3):e0264990. doi: 10.1371/journal.pone.0264990 (PMC8912205; doi:10.1371/journal.pone.0264990)

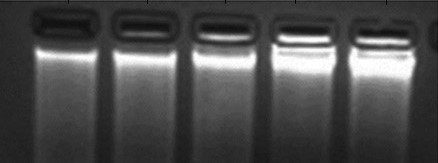

Supplement: S1 Raw image — (JPG) [file pone.0264990.s002.jpg]

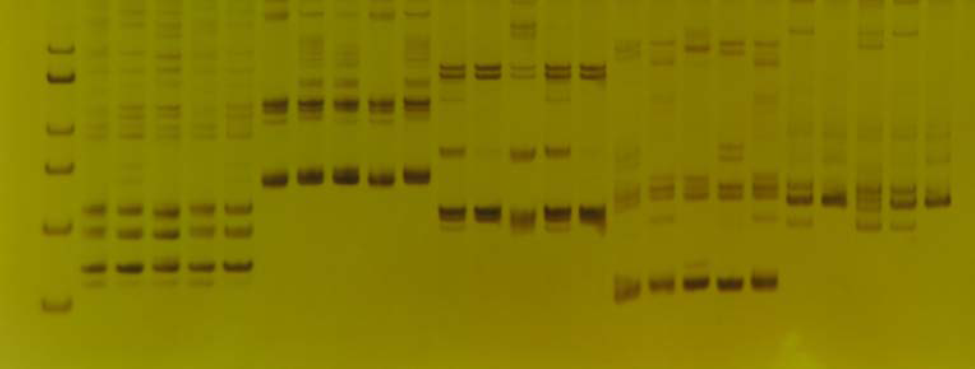

Supplement: S2 Raw image — (JPG) [file pone.0264990.s003.jpg]
